# Protocol for a randomized controlled trial: peer-to-peer Group Problem Management Plus (PM+) for adult Syrian refugees in Turkey

**DOI:** 10.1186/s13063-020-4166-x

**Published:** 2020-03-20

**Authors:** Ersin Uygun, Zeynep Ilkkursun, Marit Sijbrandij, A. Tamer Aker, Richard Bryant, Pim Cuijpers, Daniela C. Fuhr, Anne M. de Graaff, Joop de Jong, David McDaid, Naser Morina, A-La Park, Bayard Roberts, Peter Ventevogel, Taylan Yurtbakan, Ceren Acarturk, On behalf of the STRENGHTS consortium

**Affiliations:** 1 Trauma Research Laboratory, Department of Psychology, Istanbul Sehir University, Turgut Ozal Bulvari, 34865 Istanbul, Turkey; 2 Refugee Mental Health Outpatient Clinic, Bakırköy Training and Research Hospital for Mental Health and Neurological Disorders, Doktor Tevfik Saglam St, 34147 Istanbul, Turkey; 3 Department of Clinical, Neuro- and Developmental Psychology, Vrije Universiteit, Amsterdam, van der Boechorstraat 1, 1081 BT Amsterdam, The Netherlands; 4 Trauma and Disaster Mental Health, Faculty of Health Sciences, Bilgi University, Pir Hüsamettin st, 34440 Istanbul, Turkey; 5 School of Psychology, University New South Wales, Library Walk NSW, 2033 Sydney, Australia; 6 Department of Health Services Research and Policy, London School of Hygiene and Tropical Medicine, Keppel St, WC1E, 7HT London, UK; 7 Faculty of Social and Behavioral Sciences, University of Amsterdam, Nieuwe Achtergracht 166, 1001 NA Amsterdam, the Netherlands; 8 Care Policy and Evaluation Centre, Department of Health Policy, London School of Economics and Political Science, Houghton. St, WC2A 2AE London, UK; 9 University Hospital Zurich, University of Zurich, Ramistrasse 100, 8091 Zurich, Switzerland; 10 Public Health Section, United Nations High Commissioner for Refugees, Voie Creuse 5A, 1202 Geneva, Switzerland; 11 Department of Psychology, Koc University, Rumeli Feneri St Sariyer, 34450 Istanbul, Turkey

**Keywords:** Cognitive behavioural therapy, Depression, Anxiety, Post-traumatic stress, Refugee, Mental health, Group interventions, Task shifting, Trans-diagnostic

## Abstract

**Background:**

A large proportion of Syrians have been exposed to potentially traumatic events, multiple losses, and breakdown of supportive social networks and many of them have sought refuge in host countries where they also face post-migration living difficulties such as discrimination or integration problems or both. These adversities may put Syrian refugees at high risk for common mental disorders. In response to this, the World Health Organization (WHO) developed a trans-diagnostic scalable psychological intervention called Problem Management Plus (PM+) to reduce psychological distress among populations exposed to adversities. PM+ has been adapted for Syrian refugees and can be delivered by non-specialist peer lay persons in the community.

**Methods:**

A randomized controlled trial (RCT) will be conducted with 380 Syrian refugees in Turkey. After providing informed consent, participants with high levels of psychological distress (scoring above 15 on the Kessler-10 Psychological Distress Scale (K10)) and functional impairment (scoring above 16 on the WHO Disability Assessment Schedule 2.0, or WHODAS 2.0) will be randomly assigned to Group PM+/enhanced care as usual (Group PM+/E-CAU) (*n* = 190) or E-CAU (*n* = 190). Outcome assessments will take place 1 week after the fifth session (post-assessment), 3 months after the fifth session and 12 months after baseline assessment. The primary outcome is psychological distress as measured by the Hopkins Symptom Checklist (HSCL-25). Secondary outcomes include functional impairment, post-traumatic stress symptoms, self-identified problems, and health system and productivity costs. A process evaluation will be conducted to explore the feasibility, challenges and success of the intervention with 25 participants, including participants, facilitators, policy makers and mental health professionals.

**Discussion:**

The treatment manual of the Syrian-Arabic Group PM+ and training materials will be made available through the WHO once the effectiveness and cost-effectiveness of Group PM+ have been established.

**Trial registration:**

Clinical Trial Registration: ClinicalTrials.gov Identifier NCT03960892. Unique protocol ID: 10/2017. Prospectively registered on 21 May 2019.

## Background

The past eight years have resulted in a great increase of Syrian refugees, and Syria has become the principal contributor to refugees and asylum seekers worldwide [1]. A large proportion of Syrians have sought refuge in neighbouring countries such as Turkey, Jordan and Lebanon, whereas others have fled to Europe [2]. Turkey now hosts 3.6 million Syrian refugees, the largest number of Syrian refugees worldwide [1]. Syrians residing in Turkey are given the status of “under temporary protection” and not “refugee status” by the government of the Republic of Turkey. However, for simplicity, they will be called refugees throughout this article.

Similar to other refugee populations, Syrian refugees may have been exposed to potentially traumatic events such as threats to life, injuries, or witnessing deaths during the war and their flight from conflict [3, 4]. They may face post-migration difficulties such as unemployment, social isolation and discrimination after arriving in a host country [3]. Traumatic events and post-migration difficulties may make Syrian refugees more vulnerable to psychological distress and common mental disorders such as anxiety, depression and post-traumatic stress disorder (PTSD) [5–10]. If left untreated, psychological distress and mental disorders among refugees may become more profound and may create a considerable economic burden for the community [11]. In Turkey, there are currently no evidence-based community-based mental health interventions that Syrian refugees can access and that address their complex mental health needs. Moreover, Turkey lacks Arabic-speaking specialized mental health-care professionals who can deliver such treatments to Syrian refugees. In response to this treatment gap, we adapted a World Health Organization (WHO) psychological intervention called Problem Management Plus (PM+) for Syrian refugees in Turkey.

PM+ is a brief, scalable, trans-diagnostic psychological intervention that has been developed as part of the mental health Gap Action Programme [12]. PM+ addresses symptoms of depression, anxiety, PTSD and psychological distress through cognitive behavioural therapy strategies. Previous randomized controlled trials (RCTs) have demonstrated the effectiveness of individually delivered PM+ in Kenya and Pakistan and of Group PM+ in Pakistan in decreasing depression and anxiety symptoms [13–16]. In Turkey, PM+ is delivered by trained and supervised non-specialist peer refugees who have a minimum of 12 years of education. Using peer-Syrian providers as delivery agents overcomes language issues and has been found to modify the effect on patient outcomes [17]. In Turkey, Group PM+ is being used. Group counselling has been found to be as effective as individual face-to-face sessions [18]. Group sessions also reach more people at one point in time; another advantage is that patients benefit from other group members’ experiences and points of view [19].

The present RCT protocol is part of the larger Syrian Refugees Mental Health Care Systems (STRENGTHS) (https://strengths-project.eu/) [20]. STRENGTHS aims to evaluate the effectiveness and cost-effectiveness of PM+, delivered in group, individual and e-format by conducting eight RCTs in countries neighbouring Syria and in European host countries. STRENGTHS also aims to scale up the delivery and dissemination of PM+ for Syrian refugees in these countries. The PM+ group manual has been translated into Arabic and adapted for Syrian refugees and this work will be presented elsewhere. The adaptation process was carried out in accordance with the Bernal framework, which consisted of the following steps: (a) literal translation by an Arabic-speaking WHO translator, (b) free listening and key informant interviews with Syrian refugees and mental health professionals in order to identify key mental health concepts and problems among Syrian refugees and (c) cognitive testing of the translated manual [21–23].

## Objectives

The main objective of this study is to evaluate the (cost)effectiveness of the culturally adapted version of Group PM+ in adult Syrian refugees with elevated levels of psychological distress in Turkey.

### Design and setting

**Fig. 1:**
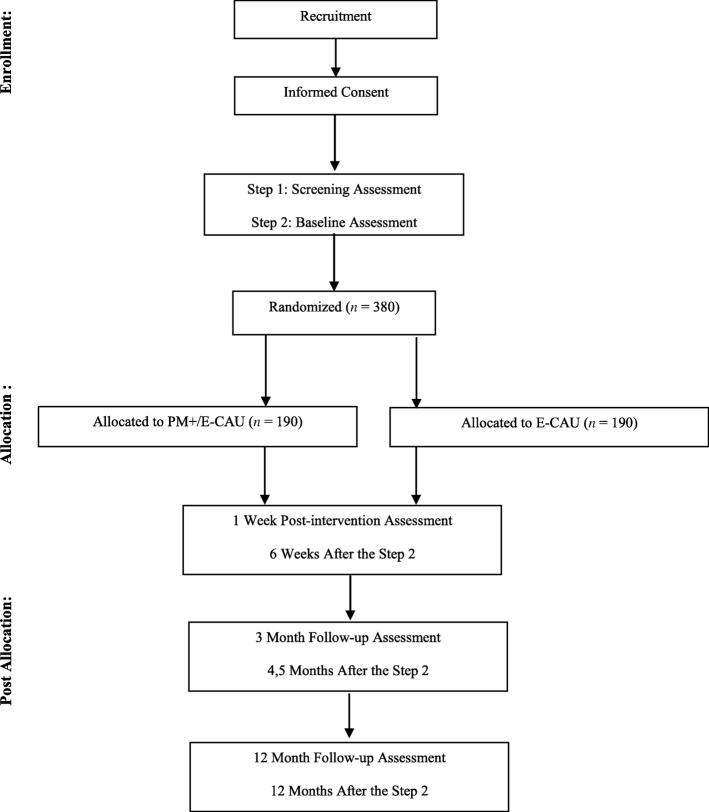
Flowchart of the study

**Fig. 2:**
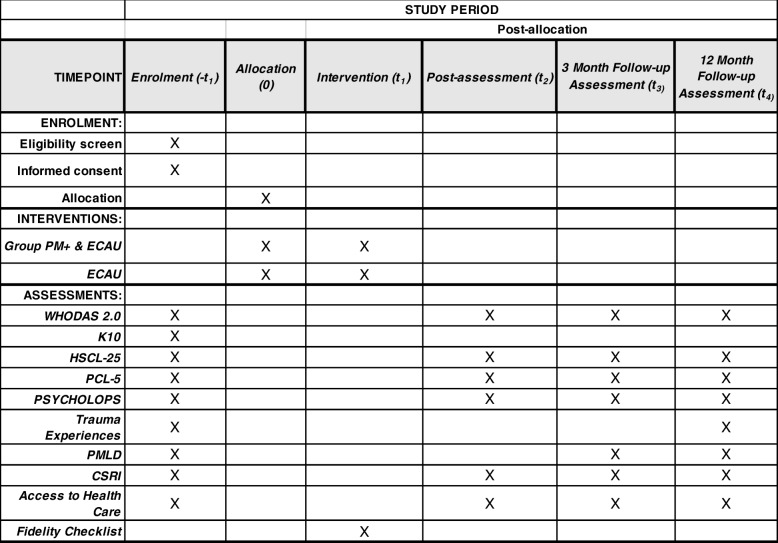
Overview of the study measures

This study is a two-arm, single-blind RCT comparing Group PM+/E-CAU with E-CAU in 380 study participants (see the flowchart in Fig. 1). It will be conducted in collaboration with the partner non-governmental organization RASASA (Refugee and Asylum Seekers Assistance and Solidarity Association) in the Sultanbeyli District of Istanbul. The Sultanbeyli District is an economically deprived area with more than 23,000 Syrians residing in overcrowded conditions in rented apartment blocks with high levels of poverty and unemployment [24]. RASASA works for the protection of refugees and provides them with psychosocial support and legal assistance. Methods of recruitment include the distribution of various advertisements and documents such as brochures and short movies in addition to social media. The study protocol has been reported in accordance with the Standard Protocol Items: Recommendations for Clinical Interventional Trials (SPIRIT) guidelines (Additional file 1). Figure 1 shows the study flowchart, and Fig. 2 shows the SPIRIT figure.

### Participant recruitment

#### Participant inclusion criteria

Participants must be adult (18 years or above) Syrians under temporary protection who are Arabic-speaking and residing in Turkey. The inclusion criteria include the following: (1) scoring greater than 16 on the WHO Disability Assessment Schedule 2.0 (WHODAS 2.0) for health and disability and (2) scoring greater than 15 on the Kessler-10 Psychological Distress Scale for psychological distress [25, 26].

#### Participant exclusion criteria

Participants will be excluded if they (1) present with an acute medical condition, (2) have an imminent risk of suicide, (3) have a severe mental disorder (e.g., psychotic disorders or substance dependence) or (4) show severe cognitive impairment (e.g., severe intellectual disability or dementia). Participants excluded from the study will be referred for appropriate treatment and support. Participants found to be eligible will be informed about their eligibility and will be invited to complete a baseline assessment before randomization (−T1).

#### Informed consent

Participants will be invited by the community RASASA for screening. Participants who attend the screening interview will be informed about the study and provided with an informed consent (IC) form which includes the right to withdraw. Participants will be informed about the use of their data and the possibility of sharing relevant data with others for research purposes. Biological specimens will not be collected in this trial. Written or witnessed oral consent (for illiterate participants) will be required before completing screening and baseline assessments.

#### Sample size and power calculations

The Vrije University (VU) University Medical Centre Department of Epidemiology and Biostatistics carried out the power calculations for this study. Considering previous studies with PM+ in Peshawar, Pakistan, and Nairobi, Kenya, we aim for a conservatively estimated small to medium Cohen’s d effect size of 0.4 in the PM+ group at the 3-month follow-up [13, 15]. Power calculations suggest a minimum sample size of 133 participants per group (power = 0.90, a = 0.05, two-sided). Considering an expected 30% attrition at the 3-month follow-up, we aim to include a total number of 380 participants (190 in the Group PM+/E-CAU group and 190 in the E-CAU group).

#### Randomization

After baseline assessment, participants will be randomly assigned on a 1:1 basis to either Group PM+/E-CAU (*n* = 190) or E-CAU (*n* = 190) (T0). Randomization using computerized software will be carried out by an independent researcher who is not involved in the intervention delivery, clinical supervision, assessments or other aspects of the study.

There is a chance that participants of the study will be recruited from the same household, creating a risk for contamination (if family members were to be allocated to receive different treatments, namely either Group PM+ or E-CAU). To limit the likelihood of contamination, participants from the same household will be assigned to the same intervention arm.

### Group PM+

PM+ is a brief, trans-diagnostic psychological intervention based on cognitive behavioural therapy techniques and developed by the WHO [12, 27]. It consists of five sessions delivered in five consecutive weeks and aims to reduce symptoms of depression, anxiety, PTSD and related conditions. Groups are separated by gender, and gender-matched facilitators will lead groups consisting of six to eight participants (T1). Sessions include evidence-based strategies of stress management, problem-solving, behavioural activation and strengthening social support. PM+ is designed to be delivered by non-specialist helpers who receive an 8-day training in PM+. These Arabic-speaking non-specialist helpers, who are called facilitators, are supervised by psychologists and psychiatrists who have participated in the PM+ training-of-trainers program. Supervision consists of weekly, face-to-face group supervision and individual supervision when needed.

### Enhanced usual care

Syrians in Turkey have free access to mental health services in public hospitals and migrant health centres [28]. However, owing to numerous barriers such as lack of knowledge of available mental health services or (self)stigma, the use of mental health services is low among Syrian refugees [29, 30]. Usual care will be enhanced by providing study participants with a leaflet on publicly available mental health services in the area where they live. Implementing Group PM+ or E-CAU will not require alteration to usual care pathways (including use of any medication) and these will continue for both trial arms.

### Outcome measures

All instruments will be administered by trained research staff blind to the allocation status of the participants at post-assessment (1 week after the fifth PM+ session) (T1), 3 months after the baseline (T2) and 12 months after baseline (T3).

#### Primary outcome measure

The primary outcome measure of this study is the Hopkins Symptom Checklist (HSCL-25). This assesses anxiety and depression symptom levels at 3-month follow-up assessment [31]. It has 25 items, namely two sub-scales for depression (13 items) and anxiety symptoms (10 items) and two items related to somatic symptoms. These items are scored on a 4-point Likert scale.

#### Secondary outcome measures

The secondary outcome measures are (1) the PTSD Checklist for DSM-5 (PCL-5) to assess the severity of post-traumatic stress reactions, (2) Psychological Outcomes Profiles (PSYCHLOPS) to assess self-identified problems, (3) the Client Service Receipt Inventory (CSRI) to assess health service utilization and productivity impacts, and (4) WHODAS, which is also part of the screening and which assesses functional disability [32, 33]. In addition, perceived access to health services will be measured (see Fig. 2). The PCL-5 has 20 items scored on a 0–4 scale, adding up to a total severity score of 80, and higher scores indicate higher levels of symptomatology. PSYCHLOPS consists of four questions on three domains: problems (two questions), function (one question) and wellbeing (one question). Problem and function questions are asked in the form of free texts, and the responses are scored on an ordinal 6-point scale, producing a maximum score of 20. The CSRI includes questions about resource use for health and social care services, informal care, and productivity as a result of functionality in people with mental disorders. The CSRI has been adapted for Syrians in Turkey to match Turkish health-care services that are available to Syrians. Relevant unit costs will be attached to service utilisation to estimate changes in use of health services and health-care costs, while Turkish minimum wage rates will be used to conservatively estimate changes in costs associated with both informal family care and lost productivity by the patient from work.

#### Other measures

A self-constructed questionnaire will be used to assess lifetime trauma exposure. This questionnaire includes items from the Harvard Trauma Questionnaire (HTQ) and the Post-traumatic Diagnostic Scale (PDS) and asks questions on specific traumatic experiences of Syrian refugees. It has 28 items scored as 0 (no) or 1 (yes), and the total score ranges from 0 to 28 [34, 35].

The Post-Migration Living Difficulties Checklist (PMLDC) will be used to assess post-migration stressors. The PMLDC has 17 items scored on a 5-point scale, ranging from 0 to 4 (0 = not a problem and 4 = very serious problem) (range 0–68). Items scored at least 2 (a moderately serious problem) are considered positive responses [36, 37].

If the validated Arabic version of the included measures is available, they will be selected and tested through cognitive interviews with Arabic-speaking adult Syrians. If the validated Arabic version of the measures is not available, these measures will be translated and back-translated in accordance with the WHO guidelines [38].

All randomly assigned participants, including those who discontinue, will be invited to all outcome assessments. In case participants do not attend a scheduled outcome assessment, they will be called a maximum of five times (on different days) to schedule a new appointment.

#### Process evaluation

We will explore the feasibility, challenges and success of the intervention through semi-structured interviews with facilitators, PM+ participants, policy makers and health professionals. From each category, five participants will be included. Interviews will take between 30 and 60 min and will start after ICs are obtained from the participants. Moreover, PM+ dose (i.e., number of sessions completed), treatment fidelity and quality will be assessed. Participant adherence to the intervention will be monitored by recording their attendance to sessions. Attendance to fewer than three sessions will be counted as dropout. Facilitators will be allowed to deliver PM+ sessions with participants after training and a competency assessment. Facilitators receive weekly supervision to ensure that intervention content is adhered to. Moreover, facilitators will be asked to complete a structured checklist on the Group PM+ components immediately after completion of each session. Trained staff members will be present during 10% of the PM+ sessions and will score treatment fidelity by using a checklist of the required PM+ elements [39].

We will promote participant retention by sending text messages to participants to remind them of their appointments. We will also regularly update phone numbers and other contact information of participants. Moreover, we offer weekend and evening hours for group sessions and outcome assessments and provide a child-friendly place for children. This may facilitate treatment access and may have a positive impact on participant retention. Participants who come to group sessions and outcome assessments will be reimbursed for travel*.*

### Data management

All data will be handled confidentially by the data monitoring committee, which consists of two researchers from Istanbul Sehir University (ISU). Quantitative data will be collected either through pen-and-paper format or through tablets. The quantitative data and the identifying key (a list connecting names to numbers) will be kept in a separate and secure locked location at the Trauma Research Lab at ISU. The data will be entered into a data-analytic computer program without the identifying key.

Qualitative interviews will be saved separately from the participants’ identifiers and will be transcribed and safely stored at the Trauma Research Lab at ISU. There will be no audio data since the Immigration Authority of Turkey does not give permission to make audio recordings in research studies with Syrian refugees (see ‘Ethics approval and consent to participate’ section).

## Statistical methods

Descriptive analyses will be carried out in Statistical Package for the Social Sciences (SPSS) and Hierarchical Linear Modelling (HLM) analyses in Stata version 11.2. Across all analyses, two-tailed tests will be reported with a *P* value of less than 0.05. To estimate the effectiveness and cost-effectiveness of Group PM+, the following data analysis methods will be conducted. First, to examine whether there are differences between conditions, *t* tests (continuous variables) or chi-squared test (categorical variables) will be conducted at baseline to compare the two intervention arms (i.e., Group PM+/E-TAU vs. E-TUA) for normally distributed data; Mann–Whitney tests will be conducted for continuous non-normally distributed data.

Intention-to-treat (ITT) analyses, including all randomly assigned participants (*N* = 380) and treatment completers’ (per protocol) analyses, will be conducted. The primary outcomes of the trial will be the ITT analyses. A linear mixed model will be used for the primary endpoint analysis to estimate the intervention effect, which will have intervention as fixed effects, baseline measurement of primary endpoint as covariate, and participants as random effects.

The mean difference between the two treatment arms at each visit/time together with its 95% confidence interval will be derived from the mixed model. A covariate-adjusted mixed model for the primary endpoint will be performed by adding pre-specified covariates at baseline (gender, age, education, traumatic experiences, post-migration difficulties, etc.) into the above model. Missing data will be treated as missing at random. No imputations of missing values will be made, as multilevel models can deal with missing data [40]. Qualitative data from the process evaluation will be analysed thematically in accordance with the framework approach [41]. Interview transcripts will be coded by using Nvivo version 11 [42].

### Economic analyses

The economic analysis will be conducted from both a health sytem perspective, and from a broader perspctive including productivity impacts in order to determine the difference in costs and outcomes in Group PM+/E-CAU, compared with E-CAU. Costs will be compared between arms using a regression model, controlling for baseline, and with bootstrapped confidence intervals using 10,000 resamples. Incremental cost-effectiveness ratios (ICERs) will be calculated comparing changes in mean costs and primary outcomes from both payer and societal perspectives between the two groups. Non-parametric bootstrapping analyses, using 10,000 resamples, will be performed to account for uncertainty and derive 95% confidence intervals around ICERs in order to generate cost-effectiveness planes. Cost-effectiveness acceptability curves will be plotted to present the probability that the PM+/E-CAU intervention is cost-effective at various willingness-to-pay threshold levels.

### Ethical considerations

The study protocol received approval from the ISU Research Ethics Committee on 12 April 2017 (Protocol ID: 12/2017) and the Immigration Authority of the Republic of Turkey to conduct the study on 29 March 2017. The STRENGTHS Safety Board (SB) will ensure that the trial and data collection are conducted in accordance with the principles of the Declaration of Helsinki (64th WMA General Assembly, Fortaleza, Brazil, October 2013). It will be in accordance with the International Conference on Harmonisation, WHO Good Clinical Practice standards, and the Medical Research Involving Human Subjects Act (WMO). There is no anticipated harm or compensation for trial participation. Cases of clinical worsening will be referred to tertiary care.

#### Trial management

The trial management committee consists of the site principal investigator (PI) and research coordinators, who are responsible for monitoring of all study procedures. Overall trial management is provided by the international PI and senior research and intervention staff, who hold monthly telephone conferences and meet annually in a face-to-face consortium meeting. The STRENGTHS SB will monitor all ethical, legal and societal issues that arise within the STRENGTHS project. In addition, the safety, rights and wellbeing of study participants and research staff will be reviewed, and interim analyses will be considered in case safety issues are (suspected to be) violated. Relevant issues will be discussed periodically (on a 6-month basis); the SB will request that additional meetings be held if issues arise in the interim.

Moreover, any amendments to the protocol will be made after a favourable opinion of the consortium and the ISU Research Ethics Committee. The coordinator of the project will then notify the funder and will update the protocol in the clinical trial registry. Any deviations from the protocol will be fully documented by using a breach report form*.*

#### Adverse event monitoring

All adverse events (AEs) and serious adverse events (SAEs) will be recorded through the web-based software, Castor Electronic Data Capture. SAEs will be reported to the ISU Research Ethics Committee within 15 days. SAEs that result in death or are life-threatening will be reported within 48 hours. All AEs will be followed until they have abated or until a stable situation has been reached.

## Discussion

This study reports the protocol of an RCT that evaluates the effectiveness and cost-effectiveness of the culturally adapted version of Group PM+ among adult Syrians residing in Turkey. PM+ is a five-session-long, trans-diagnostic psychological intervention developed by the WHO and aims to decrease symptoms of psychological distress (depression, anxiety and PTSD) and to improve psychosocial functioning.

This study builds on prior research that has demonstrated the effectiveness of PM+ in different settings in decreasing depression and anxiety symptoms [13, 15, 16]. However, PM+ has not been adapted for Syrian refugees who migrated to Europe and the Middle East after experiencing adversities in Syria, during and after migration. We hypothesise that training Arabic speakers in Group PM+ and providing peer-to-peer Group PM+ to refugees may be useful in overcoming the mental health treatment gap we are seeing in this population group. If proven to be effective, the adapted Group PM+ version will be disseminated by the WHO and recommended for scaling up.

## Trial status

The trial was registered at ClinicalTrials.gov on 21 May 2019 (ClinicalTrials.gov Identifier: NCT03960892, protocol version 4.2/201903). Recruitment of participants began on 3 August 2019 and we are planning to finish it in June 2020.

## Supplementary information

**Additional file 1.** SPIRIT (Standard Protocol Items: Recommendations for Interventional Trials) 2013 Checklist: Recommended items to address in a clinical trial protocol and related documents.

## Data Availability

The datasets analysed during this study are available from Ceren Acarturk, Koc University, Istanbul, Turkey, on reasonable request.

## Abbreviations

AE: Adverse event
CSRI: Client Service Receipt Inventory
E-CAU: Enhanced care as usual
E-TAU: Enhanced treatment as usual
IC: Informed consent ICER Cost-effectiveness ratio
ISU: Istanbul Sehir University
ITT: Intention to treat
PCL-5: PTSD Checklist for DSM-5
PI: Principal investigator
PM+: Problem Management Plus
PMLDC: Post-Migration Living Difficulties Checklist
PSYCHLOPS: Psychological Outcomes Profiles
PTSD: Post-traumatic stress disorder
RASASA: Refugee and Asylum Seekers Assistance and Solidarity Association
RCT: Randomized controlled trial
SAE: Serious adverse event
SB: Safety Board
SPIRIT: Standard Protocol Items: Recommendations for Clinical Interventional Trials
STRENGTHS: Syrian Refugees Mental Health Care Systems
WHO: World Health Organization
WHODAS: World Health Organization Disability Assessment Schedule
